# Food Additives Inhibit Intestinal Drug Transporters but Have Limited Effect on In Vitro Drug Permeability

**DOI:** 10.1021/acs.molpharmaceut.5c00705

**Published:** 2025-08-07

**Authors:** Laura Suominen, Emilia Stenberg, Noora Sjöstedt, Heidi Kidron

**Affiliations:** Drug Research Program, Division of Pharmaceutical Biosciences, Faculty of Pharmacy, 3835 University of Helsinki, Helsinki FI-00014, Finland

**Keywords:** drug transporters, beta-carotene, sulfasalazine, Caco-2, food−drug interaction

## Abstract

Food additives are chemical substances that are added to processed food to improve its flavor, texture, or appearance. Food additives can inhibit intestinal transporters, such as breast cancer resistance protein (BCRP), multidrug resistance associated protein 2 (MRP2), organic anion transporting polypeptide 2B1 (OATP2B1), and P-glycoprotein (P-gp). This inhibition could potentially affect the absorption of their substrate drugs and cause unwanted food–drug interactions. In this study, 22 food additives were evaluated for their impact on BCRP, MRP2, OATP2B1, and P-gp transport. The inhibition potency toward intestinal transporters was first studied using membrane vesicles and HEK293 cells. In these assays, four food additives (beta-carotene, butylated hydroxytoluene, dodecyl gallate, and octyl gallate) were identified as inhibitors. Seven food additives (allura red AC, beta-carotene, brilliant blue FCF, carmoisine, neohesperidin DC, sunset yellow FCF, and tartrazine), which were identified as inhibitors either in the current study or in our previous studies, were selected for Caco-2 permeability studies to further evaluate their possible effect on drug absorption. None of the selected food additives showed any effect on sulfasalazine permeability. These results suggest that the selected food additives are inhibitors of the studied transporters but are unlikely to cause clinically significant intestinal transporter-mediated drug interactions.

## Graphical abstract

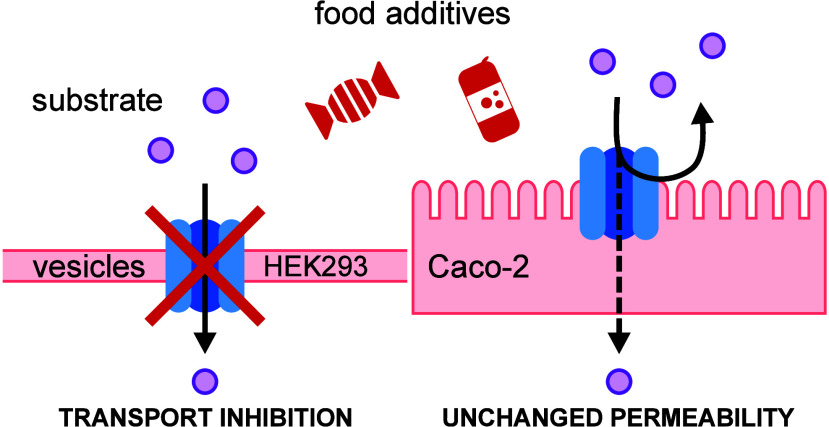

## 1 Introduction

The use of additives in processed food has increased during the recent decades, as ultraprocessed foods, which typically contain various additives, have come to predominate diets globally.^1–6^ Food additives are used to generate or improve desirable properties of food products, such as flavor, color, or texture. In the European Union (EU), over 300 substances are approved for use as food additives.^7^ Furthermore, some food additives (e.g., antioxidants) are used as pharmaceutical excipients in drug products. Pharmaceutical excipients, e.g., Tween 20, Tween 80, and PEG 400, can interact with intestinal drug transporters even though they are considered pharmacologically inactive.^8–10^ The European Food Safety Authority (EFSA) assesses the safety of food additives before they are allowed in food products in the EU, but possible metabolizing enzyme- or transporter-mediated drug interactions are rarely evaluated. We have previously studied the influence of food additives on intestinal drug transporters breast cancer resistance protein (BCRP), multidrug resistance-associated protein 2 (MRP2), organic anion transporting polypeptide 2B1 (OATP2B1), and P-glycoprotein (P-gp) in vitro. In these studies, we identified inhibitors among colorants and sweeteners.^11,12^ Others have identified transporter inhibitors among drug excipients, such as antioxidants, flavoring agents, emulsifiers, and colorants, that are also used as food additives.^13–17^

The studied transporters (BCRP, MRP2, OATP2B1, and P-gp) are important for drug absorption in the intestine since they transport a wide range of drugs through the membranes of enterocytes.^18^ P-gp, BCRP, and MRP2 belong to the family of ATP-binding cassette (ABC) transporters and are expressed in the apical membrane of enterocytes.^19–21^ Their role in enterocytes is to efflux drugs back into the gut lumen, preventing absorption. On the other hand, OATP2B1, a member of the solute carrier (SLC) transporter family, facilitates drug uptake into enterocytes where it may be expressed in both apical and basolateral membranes, although its exact localization remains uncertain.^22,23^ The interference of transporter function in the small intestine can therefore impact drug absorption and bioavailability. This transport modulation can lead to complex drug–drug interactions (DDIs) and food–drug interactions (FDIs), which are critical considerations in both clinical and dietary contexts. Regulatory agencies, such as the U.S. Food and Drug Administration (FDA) and the European Medicines Agency (EMA), recommend comprehensive studies to identify potential DDIs that could affect drug safety and efficacy as part of the drug approval process. Evaluating BCRP- and P-gp-mediated interactions is recommended, and if needed, MRP2 and OATP2B1 can be included in these studies.^24^

Studying FDIs is necessary because these interactions can be clinically significant. One of the most well-known causes of FDIs is grapefruit juice. Grapefruit juice inhibits cytochrome P450 3A4 (CYP3A4), which is the most abundant intestinal CYP enzyme.^25–27^ Thus, the bioavailability of drugs that are substrates of CYP3A4 increases.^28–33^ Grapefruit juice also inhibits the function of OATPs, and additionally, other fruit juices, such as apple and orange juice, and green tea have been found to inhibit OATPs.^34–41^ Consequently, studies should be focused not only on the inherent toxicity of food constituents but also on how they affect the disposition of endogenous compounds or xenobiotics. Moreover, unidentified FDIs may explain some of the observed interindividual variability in drug exposure.

Previous studies by us and others have shown that food additives may disturb the function of intestinal transporters. Therefore, we tested 22 additional food additives, which included sweeteners, colorants, and antioxidants. We further examined how inhibitors identified here and in our previous studies could affect drug absorption by testing their impact on the permeability of sulfasalazine, a recommended substrate for BCRP,^24^ in Caco-2 cells.

## 2 Materials and Methods

### 2.1 Materials

Unless otherwise stated, the food additives were purchased from Sigma-Aldrich (St. Louis, MO) and dissolved in dimethyl sulfoxide (DMSO) at 10–100 mM. Transporter substrates Lucifer yellow (LY), sulfasalazine, 5-carboxyfluorescein (5-CF), and 5(6)-carboxy-2,7-dichlorofluorescein (CDCF) were purchased from Sigma-Aldrich. *N*-methyl-quinidine (NMQ) was purchased from Solvo Biotechnology (Szeged, Hungary). Transporter inhibitors benzbromarone, Ko-143, and verapamil as well as all other reagents were from Sigma-Aldrich if not otherwise specified. All water used was Milli-Q water. Human embryonic kidney (HEK293) and Caco-2 cells were from the American Type Culture Collection (ATCC). Dulbecco’s modified Eagle’s medium (DMEM; cat. 32430027 for HEK293 cells and cat. 41965039 for Caco-2 cells), MEM nonessential amino acids, penicillin–streptomycin, and fetal bovine serum (FBS) were obtained from Thermo Fisher Scientific (Waltham, MA).

### 2.2 Vesicular Transport Assay

Transport inhibition by 22 food additives was tested with *Spodoptera frugiperda* (Sf9) derived crude membrane vesicles expressing BCRP, MRP2, or P-gp produced in-house. The assays were carried out as previously described in Sjöstedt et al.^11^ using fluorescent substrates, DMSO as a negative control, and positive control inhibitors in 96-well plates (Table 1). Vesicles (50 μg total protein/well) were diluted in an assay buffer (40 mM MOPS–Tris pH 7.0, 60 mM KCl, and 6 mM MgCl_2_) with the substrate, and food additives (final concentration of 50 μM) in DMSO were added to the mixture. After preincubation, either the assay buffer or Mg-ATP solution (final concentration 4 mM) was added to the plate to start the reaction. The plate was incubated at 37 °C, and the reaction was stopped with an ice-cold washing mixture (40 mM MOPS-Tris pH 7.0 and 70 mM KCl). Samples were moved to a MultiScreenHTS-FB Plate Glass fiber 1.0 μm/0.65 μm Durapore filter plate (Millipore, USA), and the wells were washed five times with the ice-cold washing mixture. For BCRP and MRP2, the fluorescent substrate was eluted from the filters with 100 μL 0.1 M NaOH. For LY samples, an equal volume of 0.1 M HCl was added to neutralize the samples before fluorescence measurement. The amount of the substrate accumulated into the vesicles was measured by fluorescent detection using a Varioskan LUX microplate reader (Thermo Fisher Scientific, Finland) (Table 1). The accumulation into the P-gp vesicles was measured with high-performance liquid chromatography (HPLC) (Agilent 110 series, Agilent Technologies, USA) after lysis and elution of the vesicle contents with 100 μL 3:1 methanol/water + 0.1% formic acid. The column used was a Poroshell 120 EC-C18 with a size of 4.6 × 100 mm and 2.7 μm particle size. Eluent A was 0.1% formic acid, and eluent B was acetonitrile. The flow rate of eluent was 1 mL/min, and the injection volume of samples was 10 μL. The following method was used for analysis: 0–1 min (15% B), 1–3 min (15–35% B), 3–4 min (90% B), and 4–6.5 min (15% B). The retention time for NMQ was around 2.5 min.

**Table 1:** Assay Conditions Used in the Inhibition Studies^a^

	substrate	positive control	preincubation time	incubation time	elution solution	fluorescence wavelengths
BCRP	LY 50 μM	sulfasalazine 50 μM	10 min	10 min	0.1 M NaOH	ex: 430 nm
						em: 538 nm
MRP2	CDCF 5 μM	benzbromarone 100 μM	10 min	10 min	0.1 M NaOH	ex: 510 nm
						em: 535 nm
P-gp	NMQ 2 μM	verapamil 100 μM	15 min	3 min	3:1 MeOH/H_2_O + 0.1% HCOOH	ex: 248 nm
						em: 442 nm
OATP2B1	5-CF 2 μM	sulfasalazine 50 μM	30 min	5 min	0.1 M NaOH	ex: 487 nm
						em: 517 nm

a 5-CF, 5-carboxyfluorescein; BCRP, breast cancer resistance protein; CDCF, 5(6)-carboxy-2,7-dichlorofluorescein; ex, excitation; em, emission; HCOOH, formic acid; LY, Lucifer yellow; MeOH, methanol; MRP2, multidrug resistance associated protein 2; NaOH, sodium hydroxide; NMQ, *N*-methyl-quinidine; OATP2B1, organic anion transporting polypeptide 2B1; and P-gp, P-glycoprotein.

### 2.3 HEK293 Uptake Assay

The inhibition of OATP2B1 was studied using a HEK293 cell-based uptake assay. HEK293 cells were cultured in DMEM supplemented with 10% FBS at 37 °C and 5% CO_2_ and seeded at 22,500 cells/well on 96-well plates coated with 0.1 mg/mL poly-d-lysine. After 24 h, the medium was replaced with a transduction mix (DMEM with 10% FBS, 5 mM sodium butyrate, and recombinant OATP2B1 or enhanced yellow fluorescent protein [eYFP, negative control] baculoviruses). The baculoviruses were produced as previously described.^12^ The uptake assay was performed after approximately 48 h. Test and preincubation solutions were prepared in a transport buffer (25 mM HEPES and 4.17 mM NaHCO_3_ in HBSS adjusted to the pH 6 with NaOH) containing the test compounds (50 μM), DMSO (0.5%), or positive control (Table 1). Test solutions also contained the substrate. Cells were washed with warm transport buffer and preincubated at 37 °C. Preincubation solutions were replaced with test solutions and incubated at 37 °C with shaking at 250 rpm. Transport was terminated by aspirating the test solution and washing the wells with an ice-cold transport buffer (150 μL) three times. Wells were left to dry, and cells were lysed with 0.1 M NaOH. Fluorescence was measured with a Varioskan LUX microplate reader (Thermo Fisher Scientific, Finland). Total protein concentration was measured from six random wells with a Pierce Coomassie (Bradford) Assay kit (Thermo Fisher Scientific) to ensure a similar protein concentration in the OATP2B1 and eYFP wells.

### 2.4 Caco-2 Permeability Assay

Beta-carotene and six other food additives (allura red AC, brilliant blue FCF, carmoisine, neohesperidin DC, sunset yellow FCF, and tartrazine) previously identified as BCRP, MRP2, or OATP2B1 inhibitors^11,12,16^ were selected for Caco-2 permeability studies to determine their potential effect on drug permeability through cell monolayers. Caco-2 cells were cultured on 12-well Transwell polycarbonate membrane inserts with a 0.4 μm pore size (Corning Incorporated, Kennebunk, ME) at 37 °C and 5% CO_2_ for 21–24 days prior to the experiment as described in Hubatsch et al.^42^ The culture medium (DMEM, 10% FBS, 1% MEM nonessential amino acids, 100 U/mL penicillin–streptomycin) was changed every second day. Transepithelial electrical resistance (TEER) was measured before and after the permeability assay with a Millicell-ERS electrode (Millipore, Bedford, MA) attached to an epithelial volt ohm meter (EVOM, World Precision Instruments, Sarasota, FL). The permeability assay was performed according to a previously published protocol.^42^ In short, the food additives, sulfasalazine (substrate), Ko-143 (BCRP control inhibitor), and LY (paracellular marker) were diluted in a transport buffer (25 mM HEPES and 4.17 mM NaHCO_3_ in HBSS adjusted to pH 7.4 with NaOH). Sulfasalazine is a substrate of BCRP and OATP2B1.^43,44^ The DMSO concentration was 0.45–1.5%. Before the experiment, inserts were washed and preincubated in the transport buffer for 20 min shaking at 37 °C. Sulfasalazine and LY permeability was measured in the apical to basolateral direction at five time points (20, 40, 60, 90, and 120 min) and, additionally, in the basolateral to apical direction to calculate the efflux ratio. Food additives (50–200 μM) were added on the apical side together with sulfasalazine (500 μM). Ko-143 (10 μM) was used on both the apical and basolateral sides to obtain maximal BRCP inhibition. LY (250 μM) was added on the donor side of all inserts to measure the integrity of the cell monolayer. Before analysis, samples were centrifuged at 2755*g* for 30 min, and donor solution and mass balance samples were diluted 1:100. LY fluorescence was measured with a Varioskan LUX microplate reader (Thermo Fisher Scientific) at 430 nm excitation and 538 nm emission. Sulfasalazine was analyzed with ultra-performance liquid chromatography (UPLC) using a Syncronis C18 column (1.7 μm particle size, 50 × 2.1 mm + 0.2 μm Waters online filter) (Thermo Fisher Scientific) coupled with Waters Acquity UPLC interfaced with a PDA detector both controlled by the Waters Empower software version 3. The UPLC was operated in gradient mode at 354 nm. Solvents were 0.015 M phosphate buffer pH 2 (A), acetonitrile (B), 1:1 acetonitrile/methanol (strong needle wash), and 20% acetonitrile (weak needle wash). Column temperature was 30 °C, injection volume was 10 μL, retention time was 1.46 min, and flow rate was 0.5 mL/min. The gradient was 25% B → 70% B (0–3 min), 70% B → 25% B (3.00–3.01 min), and 25% B (3.01–4 min). The data were collected for the first 180 s of each UPLC run.

### 2.5 Data Analysis

The ATP- or OATP2B1-dependent transport in the presence of each of the studied food additives was calculated and then compared with the corresponding transport observed for the vehicle (DMSO) control to calculate the relative transport. For the efflux transporters, the relative transport was calculated as

$$\mathrm{relative}\;\mathrm{transport}\left(\%\;\mathrm{of}\;\mathrm{control}\right)=\frac{{\mathrm{transport}}_{\mathrm{inh},\mathrm{ATP}}-{\mathrm{transport}}_{\mathrm{inh},\mathrm{no}\;\mathrm{ATP}}}{{\mathrm{transport}}_{\mathrm{DMSO},\mathrm{ATP}}-{\mathrm{transport}}_{\mathrm{DMSO},\mathrm{no}\;\mathrm{ATP}}}\times 100\%$$

where transport_inh,ATP_ and transport_inh,no ATP_ are the transport rates in the presence of food additives with or without ATP, respectively, and transport_DMSO,ATP_ and transport_DMSO,no ATP_ are the corresponding transport rates in the presence of the vehicle (DMSO) with or without ATP. For OATP2B1, the relative transport was calculated as

$$\mathrm{relative}\;\mathrm{transport}\left(\%\;\mathrm{of}\;\mathrm{control}\right)=\frac{{\mathrm{uptake}}_{\mathrm{inh},\mathrm{OATP}2B1}-{\mathrm{uptake}}_{\mathrm{inh},\mathrm{EYFP}}}{{\mathrm{uptake}}_{\mathrm{DMSO},\mathrm{OATP}2B1}-{\mathrm{uptake}}_{\mathrm{DMSO},\mathrm{EYFP}}}\times 100\%$$

where uptake_inh,OATP2B1_ and uptake_DMSO,OATP2B1_ describe the uptake of the substrate in the presence of food additives or the vehicle (DMSO), respectively, in OATP2B1 transduced cells and uptake_inh,EYFP_ and uptake_DMSO,EYFP_ describe the background passive uptake of the substrate in the presence of food additives or the vehicle (DMSO), respectively, in eYFP-transduced cells.

The half-maximal inhibitory concentrations (IC_50_) were calculated with GraphPad Prism version 9.0.2 (GraphPad Software, San Diego, CA) using nonlinear regression that fits the data to a four-parameter dose–response curve:

$$\mathrm{relative}\;\mathrm{transport}=\mathrm{minimum}+\frac{\mathrm{maximum}-\mathrm{minimum}}{1+{\left(\frac{\left[I\right]}{\mathrm{IC}50}\right)}^{h}}$$

where minimum and maximum describe the plateaus of minimal and maximal relative transport (%), [*I*] is the concentration of inhibitor, and *h* is the Hill slope, which describes the steepness of the slope.

In the Caco-2 permeability assay, the apparent permeability (*P*_app_) of sulfasalazine was calculated using the following equation:

$$P_{\mathrm{app}}=\left(\frac{dQ}{dt}\right)\left(\frac{1}{A\times C_{0}}\right)$$

where d*Q*/d*t* is the rate of permeation across the cells (nmol/min), *A* is the surface area of the filter (1.12 cm^2^), and *C*_0_ is the initial concentration in the donor chamber (μM). The efflux ratio of sulfasalazine was calculated by dividing the *P*_app_ in the basolateral to the apical direction by the *P*_app_ in the apical to basolateral direction. The TEER for each cell insert was calculated before and after the experiment by multiplying the mean resistance of three measurements by the surface area of the filter (1.12 cm^2^). Mass balance was calculated by dividing the sulfasalazine amount in the donor chamber at 0 min by the total amount of sulfasalazine at 120 min in both chambers. All experiments were done in triplicate wells, and the IC_50_ and Caco-2 permeability assays were repeated three times.

## 3 Results

### 3.1 Identification of Transporter Inhibitors

Food additives that inhibited substrate accumulation by ≥50% in single-concentration (50 μM) assays were classified as possible inhibitors. These additives were beta-carotene and three antioxidants (octyl gallate, dodecyl gallate, and butylated hydroxytoluene). Beta-carotene inhibited P-gp, BCRP, and OATP2B1 (Figure 1), and octyl gallate inhibited BCRP and OATP2B1. Dodecyl gallate inhibited only BCRP, and butylated hydroxytoluene was the only food additive that had inhibition potency toward MRP2. Other tested food additives did not inhibit the transporters or had a less than 50% inhibitory effect.

**Figure 1:**
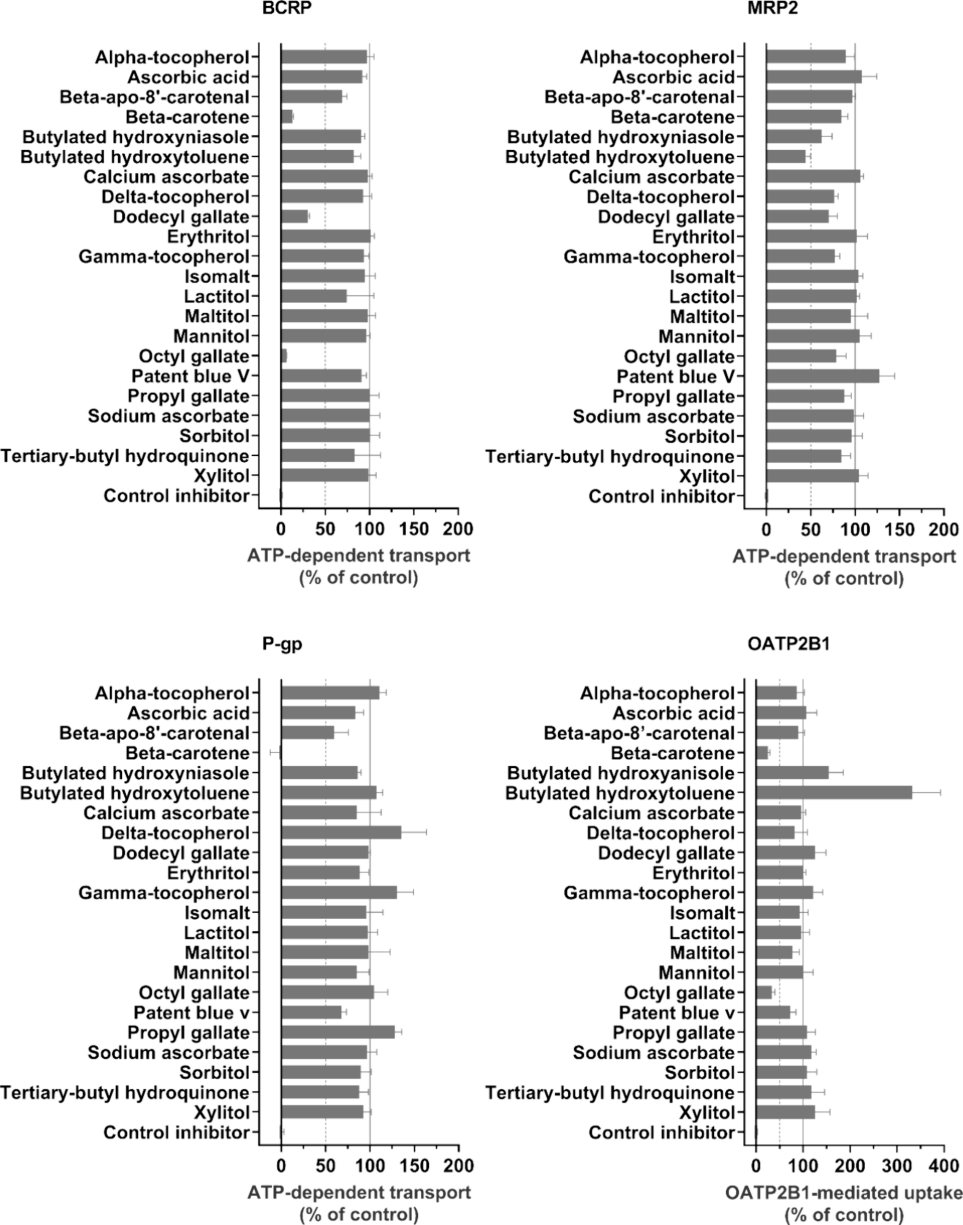
Transporter inhibition by food additives (50 μM). Inhibition of BCRP, MRP2, and P-gp transport was studied in a vesicular transport assay using Lucifer yellow (50 μM), 5(6)-carboxy-2,7-dichlorofluorescein (5 μM), and *N*-methyl-quinidine (2 μM) as substrates, respectively. Inhibition of OATP2B1-mediated uptake was studied in HEK293 cells using 5-carboxyfluorescein (2 μM) as a substrate. Results are presented as relative transport activity normalized to the control ATP-dependent transport or OATP2B1-mediated uptake (DMSO, 100%). The positive control inhibitors used were sulfasalazine (50 μM) for BCRP and OATP2B1, benzbromarone (100 μM) for MRP2, and verapamil (100 μM) for P-gp. Bars show the mean ± SD from three replicate wells; *n* = 1.

Beta-carotene, butylated hydroxytoluene, and dodecyl gallate were selected as novel inhibitors for dose–response studies to determine the IC_50_ values (Figure 2). All IC_50_ values were below 50 μM, in line with the results in Figure 1. The lowest IC_50_ value (3.2 μM) was observed for the beta-carotene inhibition of P-gp (Table 2).

**Figure 2:**
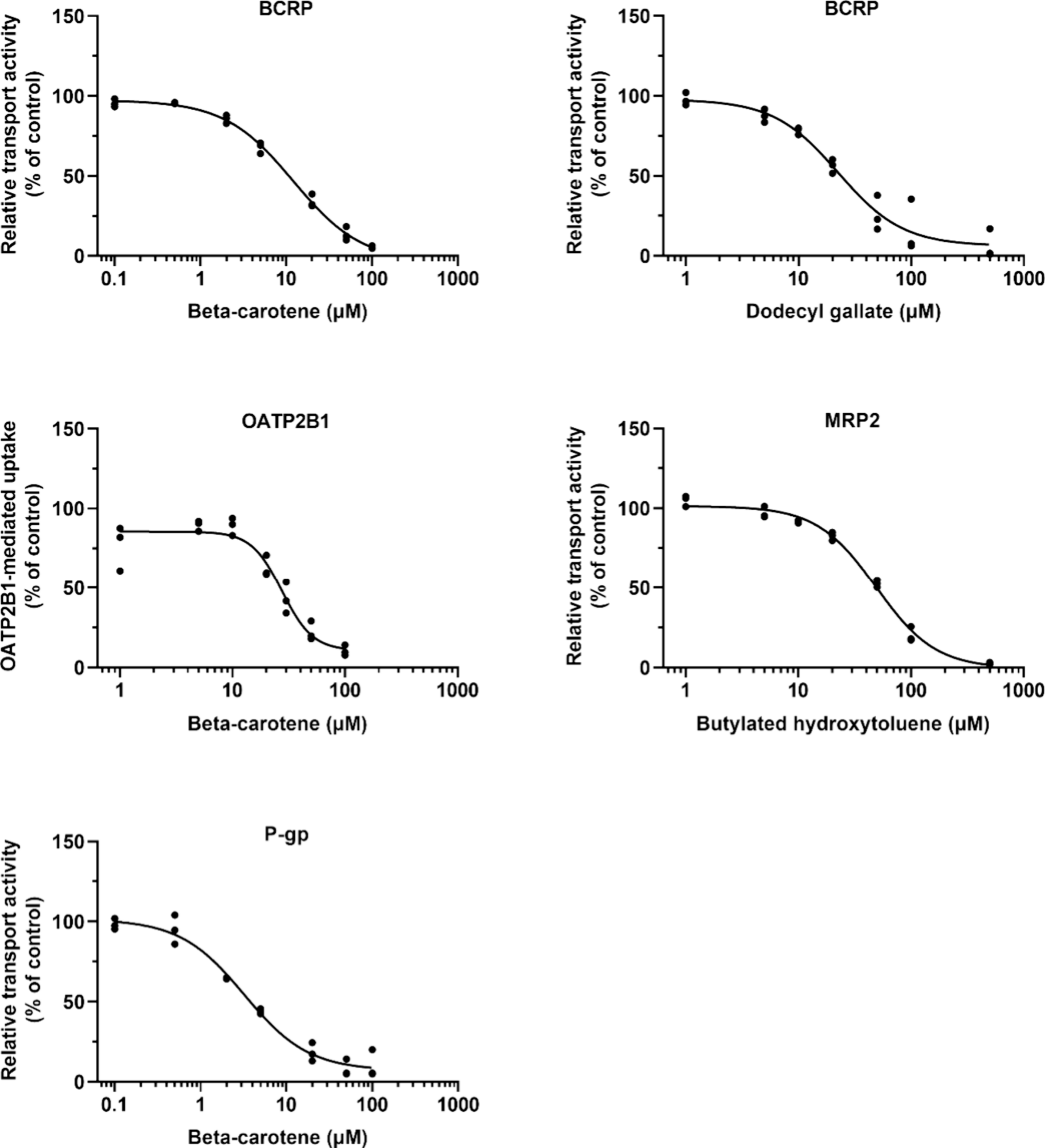
Dose–response curves of transporter inhibition by food additives. BCRP inhibition by beta-carotene and dodecyl gallate, P-gp inhibition by beta-carotene, and MRP2 inhibition by butylated hydroxytoluene were studied in a vesicular transport assay using Lucifer yellow (50 μM), *N*-methyl-quinidine (2 μM), and 5(6)-carboxy-2,7-dichlorofluorescein (5 μM) as substrates, respectively. OATP2B1 inhibition by beta-carotene was studied in HEK293 cells using 5-carboxyfluorescein (2 μM) as a substrate. Circles represent means from three independent experiments (*n* = 3) performed in triplicate wells, and the curves show nonlinear regression.

**Table 2:** Half-Maximal Inhibitory Concentrations (IC_50_) of Beta-Carotene, Butylated Hydroxytoluene, Dodecyl Gallate, and Octyl Gallate and Previously Characterized Inhibitors Selected for the Caco-2 Assay

	IC_50_ (95% CI), μM			
	BCRP	P-gp	OATP2B1	MRP2
beta-carotene	12.1 (9.9–15.5)	3.2 (2.4–4.3)	27.5 (22.6–35.6)	NA
butylated hydroxytoluene	NA	NA	NA	49.0 (43.1–55.4)
dodecyl gallate	22.5 (16.4–31.2)	NA	NA	NA
octyl gallate	10.0 (6.4–15.7)^45^	NA	ND	19.2 (14.8–24.9)^45^
allura red AC	13.3;^11^ 4.42 ^16^	NA	0.8 ^12^ ^,^ ^a^; 1.6 ^12^ ^,^ ^b^; 2.59 ^17^ ^,^ ^c^	20.0 ^11^
brilliant blue FCF	1.97 ^16^	NA	18.4 ^12^ ^,^ ^b^; 13.0 ^17^ ^,^ ^c^	3.22 ^11^
carmoisine	5.38 ^11^	NA	9.7 ^12^ ^,^ ^a^; 2.9 ^12^ ^,^ ^b^	29.0 ^11^
neohesperidin DC	0.86 ^11^	NA	48.6 ^12^ ^,^ ^a^; 15.5 ^12^ ^,^ ^b^; 20.1 ^17^ ^,^ ^c^	NA
sunset yellow FCF	10.6;^11^ 14.1 ^16^	NA	20.3 ^12^ ^,^ ^b^; 68.4 ^17^ ^,^ ^c^	NA
tartrazine	23.5;^11^ 5.61 ^16^	NA	NA	NA

a pH 7.4.

b pH 5.5.

c *K*_i_.

Assay interference studies were performed to rule out possible false inhibition results (Supporting Information). Beta-carotene interfered with the fluorescence of all four substrates by more than 20% at the highest possible concentration, and for LY and 5-CF, quenching up to ∼30% was seen also with lower beta-carotene concentrations (Figure S1). Dodecyl gallate quenched the fluorescence of LY by ≥20% at the highest concentration, but octyl gallate and butylated hydroxytoluene did not interfere with the fluorescence of any of the compounds.

### 3.2 Caco-2 Permeability Assay

To further study the inhibitory potential of food additives, the effect of seven additives on sulfasalazine permeability was studied in Caco-2 Transwell cultures. The low *P*_app_ of LY (≤0.1 × 10^–6^ cm/s), high TEER values (>300 Ω cm^2^), and high sulfasalazine efflux ratio (>70) imply that the Caco-2 cells formed a tight polarized monolayer with low paracellular permeability and efflux transporters expressed at the apical side. None of the food additives affected the *P*_app_ of sulfasalazine when added only on the apical side (Figure 3). Apical to basolateral *P*_app_ for sulfasalazine alone and with any of the food additives was ≤0.1 × 10^–6^ cm/s. The control inhibitor Ko-143 increased the permeability to ∼0.6 × 10^–6^ cm/s. For sulfasalazine alone, the basolateral to apical *P*_app_ was ∼6.5 × 10^–6^ cm/s. The inhibition potential of Ko-143 was weaker when it was applied only on the apical side (*P*_app_ fold change ≤ 2) of Caco-2 cells compared to when it was on the apical and basolateral sides (*P*_app_ fold change ≥ 5). Thus, we tested the effects of two of the food additives, tartrazine and carmoisine, when added on both sides of the cell monolayer, but the effect on sulfasalazine permeability was negligible (*P*_app_ fold change ≤ 2) (Figure S2). After the experiment, the mass balance of sulfasalazine was >90% in all wells.

**Figure 3:**
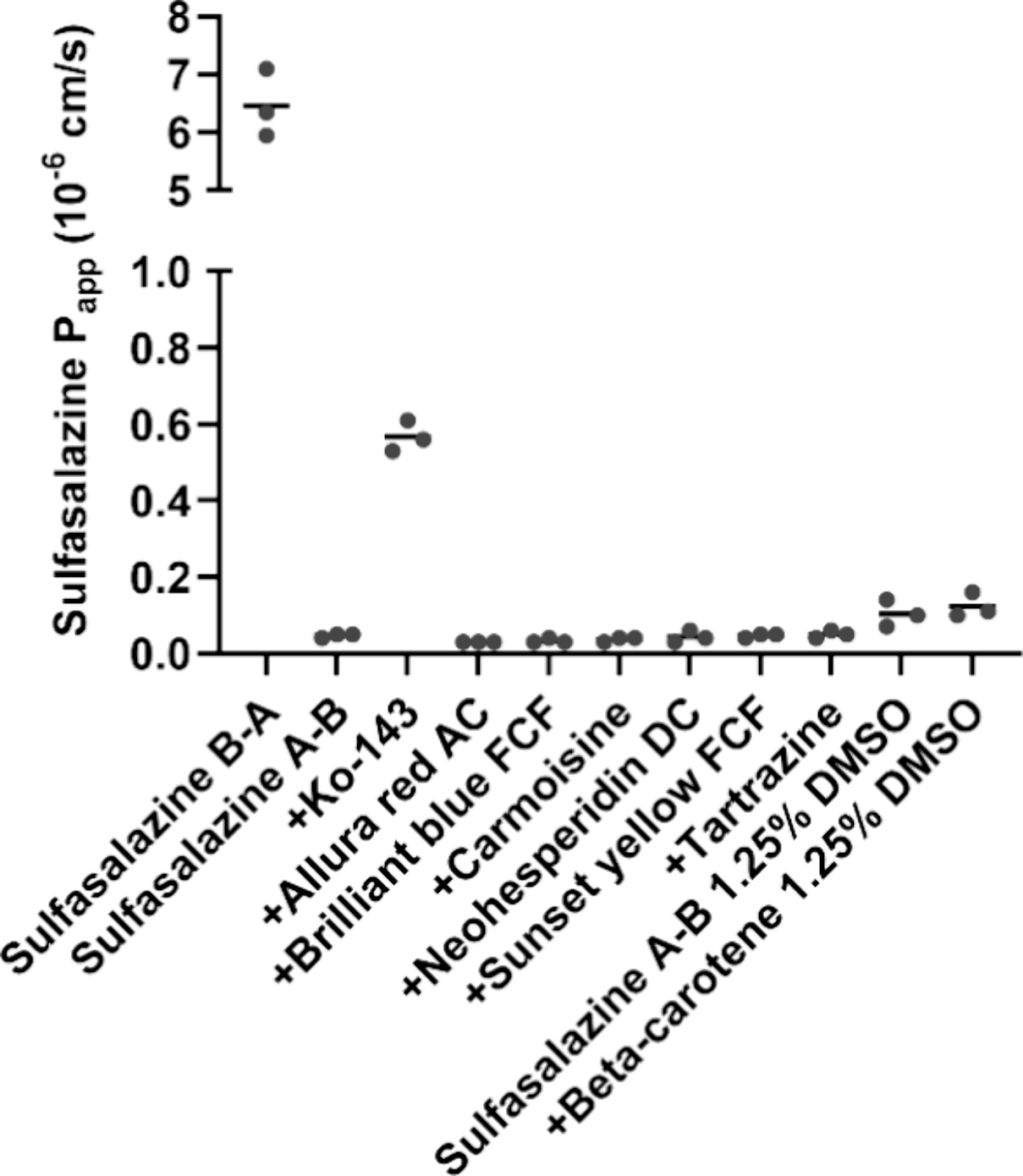
Effects of selected food additives on the sulfasalazine permeability in Caco-2 cells. The inhibition of transporters by seven food additives was studied in Caco-2 cells by measuring the apparent permeability (*P*_app_) of sulfasalazine. Food additives (200 μM, except for neohesperidin (100 μM)) were added to the apical side of the Caco-2 cell monolayer, and sulfasalazine (500 μM) permeability was measured in the apical to basolateral direction. Ko-143 (10 μM) was used as the control inhibitor. DMSO concentration was 0.45% unless otherwise stated. Circles present *P*_app_ from three replicate wells from a representative study, and lines show the mean. A–B = apical to basolateral; B–A = basolateral to apical.

## 4 Discussion

The potential of food additives to cause drug interactions is unclear. Many food additives have been identified in vitro as inhibitors of intestinal drug transporters.^11–17^ However, their effect on transcellular permeability has rarely been studied. Here, we tested the inhibitory potential of 22 food additives on BCRP, P-gp, MRP2, and OATP2B1 mediated transport and further evaluated the effects of seven food additives on sulfasalazine permeability over a Caco-2 monolayer.

In the initial testing, we found four potential inhibitors: beta-carotene, butylated hydroxytoluene, dodecyl gallate, and octyl gallate. Dose–response studies confirmed that these compounds cause 50% inhibition at ≤50 μM. Despite beta-carotene partially quenching the fluorescence signal (Figure S1), its concentration is presumed to be low during fluorescent measurement, and thus, we conclude that it is an inhibitor of BCRP, P-gp, and OATP2B1. In addition, the inhibitory activity is probably not due to membrane effects as beta-carotene did not inhibit MRP2. Previous studies support this finding of inhibition, as beta-carotene was identified as an inhibitor of P-gp in Caco-2 cells,^46^ and it significantly inhibited the transport of calcein-AM, rhodamine 123, and doxorubicin in *ABCB1*/Flp-In-293 cells,^47^ while it had only weak inhibition potency toward BCRP.^47^ Also in our study, P-gp inhibition was stronger than BCRP inhibition, but the difference was small. Interestingly, beta-apo-8’-carotenal did not inhibit these transporters as markedly as beta-carotene, although their structures are similar.

There is contradictory information about the inhibition potency of octyl gallate. In our previous study, octyl gallate inhibited BCRP and MRP2 ^45^ (Table 2). Here, octyl gallate did not inhibit MRP2 or P-gp, and for OATP2B1, only 65% inhibition was seen at 50 μM. Due to the relatively weak inhibition, we did not include OATP2B1 in octyl gallate dose–response studies. Contrary to our results, octyl gallate and dodecyl gallate inhibited the function of P-gp in KB-C2 cells.^48^

When Kulkarni et al.^15^ studied the effect of antioxidants on BCRP, OATP2B1, and P-gp, butylated hydroxytoluene did not inhibit any of the transporters, which is in line with our results. Moreover, it was observed that butylated hydroxytoluene had an OATP2B1-stimulating effect in both their and our studies. Kulkarni et al.^15^ identified butylated hydroxyanisole as a significant inhibitor of all three transporters, but the determined IC_50_ values were higher than 50 μM, especially for BCRP and P-gp. Similarly, in our study, butylated hydroxyanisole did not inhibit BCRP, OATP2B1, or P-gp more than 50% at 50 μM. Bajaj et al.^14^ studied the interaction of 123 commonly used oral molecular excipients with P-gp. None of the excipients, which were included in both our and their studies (α-tocopherol, ascorbic acid, butylated hydroxytoluene, erythritol, isomalt, lactitol, maltitol, mannitol, propyl gallate, sorbitol, and xylitol), inhibited P-gp.

Intestinal drug concentrations that rise above 10-fold their IC_50_ values are considered relevant for clinical drug interactions.^24^ To use the same cutoff value for food additives, their concentrations in food and beverages and amount of intake should be known. The daily dietary intake for beta-carotene, whose richest sources are yellow, orange, and green leafy fruits and vegetables, is estimated to be 1.46–5.84 mg.^49–53^ However, the daily intake of beta-carotene may increase considerably if a dietary supplement is used. These supplements can contain up to 100 mg of beta-carotene, which might increase its intestinal concentration to 745 μM, which is over 27-fold the IC_50_ values determined here. The concentrations of allura red, carmoisine, sunset yellow, and tartrazine in beverages can be as high as ∼850–2300 mg/L,^54,55^ and thus, their intestinal concentrations also may temporarily be high and exceed the IC_50_ values by 33–5880-fold, which would warrant further studies based on the current ICH drug interaction guidance. In the EU, up to 200 mg/L of colorants are accepted in certain beverages.^7^

In the Caco-2 assay, food additives were tested at 50–200 μM to mimic high-level consumption in the EU. Butylated hydroxytoluene, dodecyl gallate, and octyl gallate were not included in these studies since based on the determined IC_50_ values, the level of inhibition at their maximal expected intestinal concentrations is expected to be low. In addition, dodecyl gallate and octyl gallate were removed from the EU list of approved food additives in 2018 due to the lack of sufficient toxicological data.^56^

Caco-2 cells have been used to study drug permeability across cell monolayers to mimic intestinal absorption for over 20 years,^57^ and regulatory agencies recommend their use in transporter DDI studies.^24^ When grown on semipermeable membranes, Caco-2 cells form polarized cell monolayers with tight junctions and microvilli.^58^ They express most of the clinically relevant membrane transporters that are present in the intestine, but their expression levels (e.g., OATP2B1) may be higher in Caco-2 cells.^59–61^ BCRP, P-gp, and MRP2 are expressed on the apical membranes of Caco-2 cells,^62–64^ but OATP2B1 may be expressed on both membranes as in the intestine.^23,65,66^

Sulfasalazine is a high-affinity substrate of BCRP and OATP2B1.^43,44^ We selected it as a substrate for the Caco-2 assay due to its low apical to basolateral permeability in vitro and low intestinal absorption in humans, both of which increase upon BCRP inhibition or dysfunction.^67,68^ The ICH drug interaction guideline lists sulfasalazine as an in vitro and clinical substrate of BCRP, encouraging its use in drug interaction studies.^24^ Here, sulfasalazine permeability was measured in the apical to basolateral direction to mimic intestinal absorption. At 10 μM, Ko-143 might inhibit MRP2, OATP2B1, and P-gp even though the inhibitory effect should be strongest for BCRP (IC_50_ < 0.5 μM).^44,69–71^ In addition, most of the compounds studied inhibit both BCRP and OATP2B1 in the overexpression systems (Table 2). This considerable overlap between transporter substrates and inhibitors might complicate the interpretation of Caco-2 assay results. For example, the inhibition of apical BCRP should increase the apical to basolateral permeability of sulfasalazine, while the inhibition of apical OATP2B1 could decrease it. Inhibition of both BCRP and OATP2B1 may show as unchanged permeability, which could occur in our assay for several additives. However, sulfasalazine permeability was unchanged even in the presence of tartrazine, which only inhibits BCRP. Moreover, considering the high efflux ratio of >70 observed for sulfasalazine (and assuming a primarily apical expression of OATP2B1), BCRP is expected to be the main transporter of sulfasalazine in our Caco-2 cells. The influence of BCRP is also supported by the sensitivity to Ko-143, which is a stronger inhibitor for BCRP than for OATP2B1. Therefore, we assume that changes in sulfasalazine permeability would be primarily mediated by BCRP inhibition. Interestingly, the inhibition potential of Ko-143 was stronger when it was applied on the basolateral side compared to the apical side (Figure S2), suggesting a more permeable basolateral side. Despite the complex interpretation of sulfasalazine permeability in the Caco-2 assay, membrane transporters expressed in Caco-2 cells are expressed also in the intestine,^59^ which support the relevance of Caco-2 data.

Differences between the vesicular transport (Table 2) and Caco-2 assay results (Figure 3) could be explained by the low permeability of the food additives. The vesicular transport assay may overestimate the inhibitory potential of water-soluble compounds, such as synthetic food dyes, since the intracellular side of the transporter is facing outward and can directly interact with extravesicular compounds. In Caco-2 cells, if the transporter interaction site is intracellular, e.g., the ATP-binding site or the substrate binding cavity, compounds have to permeate through the cell membrane before binding to the transporter. Allura red AC, brilliant blue FCF, carmoisine, sunset yellow FCF, and tartrazine are water-soluble (LogD_7.4_ < 0), and according to EFSA’s evaluations, their in vivo absorption is expected to be low.^72–76^ Thus, they are likely unable to permeate through cell membranes to inhibit efflux transporters. Inhibition of uptake transporters is, however, possible as Zou et al.^17^ found that 25 mg/kg allura red AC inhibits OATP2B1 and decreases fexofenadine plasma levels in P-gp-deficient mice.

Beta-carotene and neohesperidin DC are more lipophilic (LogD_7.4_ = 13.6 and 2.6, respectively) than the five colorants and could thus more readily partition into the cell membrane. This has been seen in Caco-2 cells where the absorption efficiency of beta-carotene from a micellar formulation into cells was up to ∼11%, and up to ∼35% of the applied beta-carotene dose was recovered in the basolateral chamber.^77,78^ Also, neohesperidin DC had moderate permeability (*P*_app_ < 6 × 10^–6^ cm/s) in Caco-2 cells,^79^ and in rats, its oral bioavailability was ∼20%.^80^ Despite the better permeability, the lipophilicity of neohesperidin DC and beta-carotene can cause solubility problems in aqueous assay buffers. Their aggregation may cause nonspecific inhibition^81^ or prevent uptake into cells. In our assay, the DMSO stocks of food additives were diluted in the buffer and applied to Caco-2 cells. Micellization could have improved the solubility and uptake of beta-carotene.

Another possible explanation for the unchanged sulfasalazine permeability is the lack of preincubation with food additives. It is known that adding a preincubation step to inhibition studies of SLC transporters can increase the inhibitory potential compared to the coincubation method,^82,83^ and hence, we used preincubation in the OATP2B1 inhibition studies in HEK293 cells. The proposed mechanism for the preincubation effect is that the inhibitor has enough time to reach the intracellular interaction side of transporters.^82,84^ In the Caco-2 assay, the coincubation time with the inhibitor and substrate is considerably longer (i.e., 120 min) than in SLC uptake assays, e.g., in HEK293 cells (2–10 min). This long coincubation time may facilitate the permeation of the inhibitor and thus reduce the impact of preincubation.^82,85^ However, it would be interesting to study the preincubation effect also in Caco-2 cells since food additives are not always consumed together with drugs.

In addition to the dose and time of administration, other factors affect the inhibitory potential of food additives in humans. For example, human intestinal microflora can metabolize food additives such as sweeteners and azo dyes,^86–88^ which could diminish their inhibitory effects. Zou et al.^17^ hypothesize, however, that high concentrations of azo dyes in the intestine might be able to saturate azoreductase capacity of gut microbiome, which also highlights the importance of predicting the intestinal concentrations of food additives. Nevertheless, it should be noted that the concentration inside the enterocyte can differ from the intestinal concentration. The intracellular concentration is especially important when considering the function of efflux transporters since their interaction site is usually intracellular.^89,90^ Hence, more in vivo and clinical studies are needed to fully understand the effects of food additives. A final limitation of our inhibition studies may be that transporter inhibition can be substrate-dependent. For example, it is known that P-gp has multiple binding sites,^91,92^ and thus, inhibition of substrate transport does not fully reflect the inhibitory ability of the food additive if its interaction site is different from that of the used substrate. Since only one substrate was used in the initial assays (Table 1), our results do not definitively rule out the possibility of inhibition.

## 5 Conclusions

From the 22 tested food additives, four (beta-carotene, butylated hydroxytoluene, dodecyl gallate, and octyl gallate) inhibit intestinal drug transporters in vitro in overexpression systems. However, when seven food additives with inhibitory effects toward intestinal drug transporters were tested in Caco-2 cells, their effect on sulfasalazine permeability was negligible. These results suggest that even though many food additives can inhibit drug transporters, they might have limited effect on drug permeability and absorption from the intestine.

## Supplementary Material



## Notes

Results from fluorescent interaction studies and effect of Ko-143 on sulfasalazine permeability from the apical, basolateral, or apical and basolateral side of Caco-2 cells (PDF)

## References

[1] Baker P., Machado P., Santos T., Sievert K., Backholer K., Hadjikakou M., Russell C., Huse O., Bell C., Scrinis G., Worsley A., Friel S., Lawrence M. (2020). Ultra-Processed
Foods and the Nutrition Transition: Global, Regional and National
Trends, Food Systems Transformations and Political Economy Drivers. Obes. Rev..

[2] Chazelas E., Deschasaux M., Srour B., Kesse-Guyot E., Julia C., Alles B., Druesne-Pecollo N., Galan P., Hercberg S., Latino-Martel P., Esseddik Y., Szabo F., Slamich P., Gigandet S., Touvier M. (2020). Food Additives: Distribution and Co-Occurrence in 126,000
Food Products of the French Market. Sci. Rep..

[3] Dunford E. K., Miles D. R., Popkin B. (2023). Food Additives
in Ultra-Processed
Packaged Foods: An Examination of US Household Grocery Store Purchases. J. Acad. Nutr. Diet..

[4] Monteiro C. A., Cannon G., Levy R. B., Moubarac J.-C., Louzada M. L., Rauber F., Khandpur N., Cediel G., Neri D., Martinez-Steele E., Baraldi L. G., Jaime P. C. (2019). Ultra-Processed
Foods: What They Are and How to Identify Them. Public Health Nutr..

[5] Moubarac J.-C., Batal M., Louzada M. L., Martinez
Steele E., Monteiro C. A. (2017). Consumption of Ultra-Processed Foods
Predicts Diet
Quality in Canada. Appetite.

[6] Slimani N., Deharveng G., Southgate D. A. T., Biessy C., Chajès V., van Bakel M. M. E., Boutron-Ruault M. C., McTaggart A., Grioni S., Verkaik-Kloosterman J., Huybrechts I., Amiano P., Jenab M., Vignat J., Bouckaert K., Casagrande C., Ferrari P., Zourna P., Trichopoulou A., Wirfält E., Johansson G., Rohrmann S., Illner A. K., Barricarte A., Rodríguez L., Touvier M., Niravong M., Mulligan A., Crowe F., Ocké M. C., van der Schouw Y. T., Bendinelli B., Lauria C., Brustad M., Hjartåker A., Tjønneland A., Jensen A. M., Riboli E., Bingham S. (2009). Contribution of Highly Industrially Processed Foods
to the Nutrient Intakes and Patterns of Middle-Aged Populations in
the European Prospective Investigation into Cancer and Nutrition Study. Eur. J. Clin. Nutr..

[7] Food and feed information portal database version 5.3. European Comission Food and Feed Information Portal Database. Based on Annex II of Regulation (EC) No 1333/2008. https://ec.europa.eu/food/food-feed-portal/screen/food-additives/search (accessed 2025–02–20).

[8] Engel A., Oswald S., Siegmund W., Keiser M. (2012). Pharmaceutical Excipients
Influence the Function of Human Uptake Transporting Proteins. Mol. Pharmaceutics.

[9] Hanke U., May K., Rozehnal V., Nagel S., Siegmund W., Weitschies W. (2010). Commonly Used
Nonionic Surfactants Interact Differently with the Human Efflux Transporters
ABCB1 (p-Glycoprotein) and ABCC2 (MRP2). Eur.
J. Pharm. Biopharm..

[10] Yamagata T., Morishita M., Kusuhara H., Takayama K., Benameur H., Sugiyama Y. (2009). Characterization of the Inhibition
of Breast Cancer
Resistance Protein-Mediated Efflux of Mitoxantrone by Pharmaceutical
Excipients. Int. J. Pharm..

[11] Sjöstedt N., Deng F., Rauvala O., Tepponen T., Kidron H. (2017). Interaction
of Food Additives with Intestinal Efflux Transporters. Mol. Pharmaceutics.

[12] Tikkanen A., Pierrot E., Deng F., Sánchez V. B., Hagström M., Koenderink J. B., Kidron H. (2020). Food Additives as Inhibitors
of Intestinal Drug Transporter OATP2B1. Mol.
Pharmaceutics.

[13] Al-Ali A. A. A., Nielsen R. B., Steffansen B., Holm R., Nielsen C. U. (2019). Nonionic
Surfactants Modulate the Transport Activity of ATP-Binding Cassette
(ABC) Transporters and Solute Carriers (SLC): Relevance to Oral Drug
Absorption. Int. J. Pharm..

[14] Bajaj R., Chong L. B., Zou L., Tsakalozou E., Ni Z., Giacomini K. M., Kroetz D. L. (2021). Interaction of Commonly Used Oral
Molecular Excipients with P-Glycoprotein. AAPS
J..

[15] Kulkarni C. P., Yang J., Koleske M. L., Lara G., Alam K., Raw A., Rege B., Zhao L., Lu D., Zhang L., Yu L. X., Lionberger R. A., Giacomini K. M., Kroetz D. L., Yee S. W. (2024). Effect
of Antioxidants in Medicinal
Products on Intestinal Drug Transporters. Pharmaceutics.

[16] Zou L., Pottel J., Khuri N., Ngo H. X., Ni Z., Tsakalozou E., Warren M. S., Huang Y., Shoichet B. K., Giacomini K. M. (2020). Interactions
of Oral Molecular Excipients with Breast
Cancer Resistance Protein. BCRP. Mol. Pharm..

[17] Zou L., Spanogiannopoulos P., Pieper L. M., Chien H.-C., Cai W., Khuri N., Pottel J., Vora B., Ni Z., Tsakalozou E., Zhang W., Shoichet B. K., Giacomini K. M., Turnbaugh P. J. (2020). Bacterial Metabolism Rescues the Inhibition of Intestinal
Drug Absorption by Food and Drug Additives. Proc. Natl. Acad. Sci. U. S. A..

[18] Iversen D. B., Andersen N. E., Dalgård
Dunvald A.-C., Pottegård A., Stage T. B. (2022). Drug Metabolism
and Drug Transport of the 100 Most
Prescribed Oral Drugs. Basic Clin. Pharmacol.
Toxicol..

[19] Fromm M. F., Kauffmann H.-M., Fritz P., Burk O., Kroemer H. K., Warzok R. W., Eichelbaum M., Siegmund W., Schrenk D. (2000). The Effect
of Rifampin Treatment on Intestinal Expression of Human MRP Transporters. Am. J. Pathol..

[20] Maliepaard M., Scheffer G. L., Faneyte I. F., van Gastelen M. A., Pijnenborg A. C., Schinkel A. H., van De
Vijver M. J., Scheper R. J., Schellens J. H. (2001). Subcellular
Localization and Distribution
of the Breast Cancer Resistance Protein Transporter in Normal Human
Tissues. Cancer Res..

[21] Thiebaut F., Tsuruo T., Hamada H., Gottesman M. M., Pastan I., Willingham M. C. (1987). Cellular
Localization of the Multidrug-Resistance
Gene Product P-Glycoprotein in Normal Human Tissues. Proc. Natl. Acad. Sci. U. S. A..

[22] Keiser M., Kaltheuner L., Wildberg C., Müller J., Grube M., Partecke L. I., Heidecke C.-D., Oswald S. (2017). The Organic
Anion-Transporting Peptide 2B1 Is Localized in the Basolateral Membrane
of the Human Jejunum and Caco-2 Monolayers. J. Pharm. Sci..

[23] Kobayashi D., Nozawa T., Imai K., Nezu J., Tsuji A., Tamai I. (2003). Involvement of Human
Organic Anion Transporting Polypeptide OATP-B
(SLC21A9) in pH-Dependent Transport across Intestinal Apical Membrane. J. Pharmacol. Exp. Ther..

[24] ICH . ICH Harmonised Guideline Drug Interaction Studies M12 - International Council for Harmonisation of Technical Requirements for Pharmaceuticals for Human Use. ICH 2024.

[25] Bailey D.
G., Malcolm J., Arnold O., David Spence J. (1998). Grapefruit
Juice-Drug Interactions. Br. J. Clin. Pharmacol..

[26] Drozdzik M., Busch D., Lapczuk J., Müller J., Ostrowski M., Kurzawski M., Oswald S. (2018). Protein Abundance of
Clinically Relevant Drug-Metabolizing Enzymes in the Human Liver and
Intestine: A Comparative Analysis in Paired Tissue Specimens. Clin. Pharmacol. Ther..

[27] Paine M. F., Hart H. L., Ludington S. S., Haining R. L., Rettie A. E., Zeldin D. C. (2006). The Human Intestinal
Cytochrome P450 “Pie”. Drug Metab.
Dispos. Biol. Fate Chem..

[28] Ducharme M. P., Warbasse L. H., Edwards D. J. (1995). Disposition
of Intravenous and Oral
Cyclosporine after Administration with Grapefruit Juice. Clin. Pharmacol. Ther..

[29] Fuhr U., Müller-Peltzer H., Kern R., Lopez-Rojas P., Jünemann M., Harder S., Staib H. A. (2002). Effects of Grapefruit
Juice and Smoking on Verapamil Concentrations in Steady State. Eur. J. Clin. Pharmacol..

[30] Kupferschmidt H. H. T., Ha H. R., Ziegler W. H., Meier P. J., Krähenbühl S. (1995). Interaction
between Grapefruit Juice and Midazolam in Humans. Clin. Pharmacol. Ther..

[31] Lown K. S., Bailey D. G., Fontana R. J., Janardan S. K., Adair C. H., Fortlage L. A., Brown M. B., Guo W., Watkins P. B. (1997). Grapefruit
Juice Increases Felodipine Oral Availability in Humans by Decreasing
Intestinal CYP3A Protein Expression. J. Clin.
Invest..

[32] Veronese M. L., Gillen L. P., Burke J. P., Dorval E. P., Hauck W. W., Pequignot E., Waldman S. A., Greenberg H. E. (2003). Exposure-Dependent
Inhibition of Intestinal and Hepatic CYP3A4 In Vivo by Grapefruit
Juice. J. Clin. Pharmacol..

[33] Weber A., Jäger R., Börner A., Klinger G., Vollanth R., Matthey K., Balogh A. (1996). Can Grapefruit Juice Influence Ethinylestradiol
Bioavailability?. Contraception.

[34] Abdelkawy K. S., Abdelaziz R. M., Abdelmageed A. M., Donia A. M., El-Khodary N. M. (2020). Effects
of Green Tea Extract on Atorvastatin Pharmacokinetics in Healthy Volunteers. Eur. J. Drug Metab. Pharmacokinet..

[35] Akamine Y., Miura M., Komori H., Saito S., Kusuhara H., Tamai I., Ieiri I., Uno T., Yasui-Furukori N. (2014). Effects of
One-Time Apple Juice Ingestion on the Pharmacokinetics of Fexofenadine
Enantiomers. Eur. J. Clin. Pharmacol..

[36] Jeon H., Jang I., Lee S., Ohashi K., Kotegawa T., Ieiri I., Cho J., Yoon S. H., Shin S., Yu K., Lim K. S. (2013). Apple Juice Greatly Reduces Systemic Exposure to Atenolol. Br. J. Clin. Pharmacol..

[37] Knop J., Misaka S., Singer K., Hoier E., Müller F., Glaeser H., König J., Fromm M. F. (2015). Inhibitory Effects
of Green Tea and (−)-Epigallocatechin Gallate on Transport
by OATP1B1, OATP1B3, OCT1, OCT2, MATE1, MATE2-K and P-Glycoprotein. PLoS One.

[38] Lilja J. J., Raaska K., Neuvonen P. J. (2005). Effects
of Orange Juice on the Pharmacokinetics
of Atenolol. Eur. J. Clin. Pharmacol..

[39] Tapaninen T., Neuvonen P. J., Niemi M. (2011). Orange and Apple Juice
Greatly Reduce
the Plasma Concentrations of the OATP2B1 Substrate Aliskiren. Br. J. Clin. Pharmacol..

[40] Dresser G. K., Bailey D. G., Leake B. F., Schwarz U. I., Dawson P. A., Freeman D. J., Kim R. B. (2002). Fruit Juices
Inhibit Organic Anion
Transporting Polypeptide-Mediated Drug Uptake to Decrease the Oral
Availability of Fexofenadine. Clin. Pharmacol.
Ther..

[41] Satoh H., Yamashita F., Tsujimoto M., Murakami H., Koyabu N., Ohtani H., Sawada Y. (2005). Citrus Juices Inhibit the Function
of Human Organic Anion-Transporting Polypeptide OATP-B. Drug Metab. Dispos. Biol. Fate Chem..

[42] Hubatsch I., Ragnarsson E. G. E., Artursson P. (2007). Determination of Drug Permeability
and Prediction of Drug Absorption in Caco-2 Monolayers. Nat. Protoc..

[43] Kusuhara H., Furuie H., Inano A., Sunagawa A., Yamada S., Wu C., Fukizawa S., Morimoto N., Ieiri I., Morishita M., Sumita K., Mayahara H., Fujita T., Maeda K., Sugiyama Y. (2012). Pharmacokinetic Interaction Study of Sulphasalazine
in Healthy Subjects and the Impact of Curcumin as an in Vivo Inhibitor
of BCRP. Br. J. Pharmacol..

[44] Sáfár Z., Kecskeméti G., Molnár J., Kurunczi A., Szabó Z., Janáky T., Kis E., Krajcsi P. (2021). Inhibition of ABCG2/BCRP-Mediated
Transport-Correlation Analysis of Various Expression Systems and Probe
Substrates. Eur. J. Pharm. Sci..

[45] Sjöstedt N., Holvikari K., Tammela P., Kidron H. (2017). Inhibition of Breast
Cancer Resistance Protein and Multidrug Resistance Associated Protein
2 by Natural Compounds and Their Derivatives. Mol. Pharmaceutics.

[46] Eid S. Y., El-Readi M. Z., Wink M. (2012). Carotenoids
Reverse Multidrug Resistance
in Cancer Cells by Interfering with ABC-Transporters. Phytomedicine.

[47] Teng Y.-N., Sheu M.-J., Hsieh Y.-W., Wang R.-Y., Chiang Y.-C., Hung C.-C. (2016). β-Carotene
Reverses Multidrug Resistant Cancer
Cells by Selectively Modulating Human P-Glycoprotein Function. Phytomedicine.

[48] Kitagawa S., Nabekura T., Kamiyama S., Takahashi T., Nakamura Y., Kashiwada Y., Ikeshiro Y. (2005). Effects of Alkyl Gallates
on P-Glycoprotein Function. Biochem. Pharmacol..

[49] Beltrán-de-Miguel B., Estévez-Santiago R., Olmedilla-Alonso B. (2015). Assessment
of Dietary Vitamin A Intake (Retinol, α-Carotene, β-Carotene,
β-Cryptoxanthin) and Its Sources in the National Survey of Dietary
Intake in Spain (2009–2010). Int. J.
Food Sci. Nutr..

[50] Curran-Celentano J., Hammond B. R., Ciulla T. A., Cooper D. A., Pratt L. M., Danis R. B. (2001). Relation between Dietary Intake,
Serum Concentrations,
and Retinal Concentrations of Lutein and Zeaxanthin in Adults in a
Midwest Population. Am. J. Clin. Nutr..

[51] George S. M., Thompson F. E., Midthune D., Subar A. F., Berrigan D., Schatzkin A., Potischman N. (2012). Strength of the Relationships between
Three Self-Reported Dietary Intake Instruments and Serum Carotenoids:
The Observing Energy and Protein Nutrition (OPEN) Study. Public Health Nutr..

[52] Lucarini M., Lanzi S., D’Evoli L., Aguzzi A., Lombardi-Boccia G. (2006). Intake of
Vitamin A and Carotenoids from the Italian Population – Results
of an Italian Total Diet Study. Int. J. Vitam.
Nutr. Res..

[53] O’Neill M. E., Carroll Y., Corridan B., Olmedilla B., Granado F., Blanco I., Berg H. V. D., Hininger I., Rousell A.-M., Chopra M., Southon S., Thurnham D. I. (2001). A European
Carotenoid Database to Assess Carotenoid Intakes and Its Use in a
Five-Country Comparative Study. Br. J. Nutr..

[54] Dixit S., Purshottam S. K., Gupta S. K., Khanna S. K., Das M. (2010). Usage Pattern
and Exposure Assessment of Food Colours in Different Age Groups of
Consumers in the State of Uttar Pradesh. India.
Food Addit. Contam. Part A.

[55] Husain A., Sawaya W., Al-Omair A., Al-Zenki S., Al-Amiri H., Ahmed N., Al-Sinan M. (2006). Estimates
of Dietary Exposure of
Children to Artificial Food Colours in Kuwait. Food Addit. Contam..

[56] Regulation (EC) 2018/1481. Comission Regulation (EU) 2018/1481 of 4 October 2018 Amending Annexes II and III to Regulation (EC) No 1333/2008 of the European Parliament and of the Council and the Annex to Commission Regulation (EU) No 231/2012 as Regards Octyl Gallate (E 311) and Dodecyl Gallate (E 312);&nbsp;European Union 2018.

[57] Artursson P., Palm K., Luthman K. (2001). Caco-2 Monolayers in Experimental
and Theoretical Predictions of Drug Transport1. Adv. Drug Delivery Rev..

[58] Hidalgo I. J., Raub T. J., Borchardt R. T. (1989). Characterization
of the Human Colon
Carcinoma Cell Line (Caco-2) as a Model System for Intestinal Epithelial
Permeability. Gastroenterology.

[59] Brück S., Strohmeier J., Busch D., Drozdzik M., Oswald S. (2017). Caco-2 Cells
– Expression, Regulation and Function of Drug Transporters
Compared with Human Jejunal Tissue. Biopharm.
Drug Dispos..

[60] Ölander M., Wiśniewski J. R., Matsson P., Lundquist P., Artursson P. (2016). The Proteome of Filter-Grown Caco-2 Cells With a Focus
on Proteins Involved in Drug Disposition. J.
Pharm. Sci..

[61] Uchida Y., Ohtsuki S., Kamiie J., Ohmine K., Iwase R., Terasaki T. (2015). Quantitative Targeted Absolute Proteomics
for 28 Human
Transporters in Plasma Membrane of Caco-2 Cell Monolayer Cultured
for 2, 3, and 4 Weeks. Drug Metab. Pharmacokinet..

[62] Belliard A.-M., Lacour B., Farinotti R., Leroy C. (2004). Effect of Tumor Necrosis
Factor-α and Interferon-γ on Intestinal P-Glycoprotein
Expression, Activity, and Localization in Caco-2 Cells. J. Pharm. Sci..

[63] Tocchetti G. N., Arias A., Arana M. R., Rigalli J. P., Domínguez C. J., Zecchinati F., Ruiz M. L., Villanueva S. S. M., Mottino A. D. (2018). Acute Regulation
of Multidrug Resistance-Associated
Protein 2 Localization and Activity by cAMP and Estradiol-17β-d-Glucuronide
in Rat Intestine and Caco-2 Cells. Arch. Toxicol..

[64] Xia C. Q., Liu N., Yang D., Miwa G., Gan L.-S. (2005). Expression, Localization,
and Functional Characteristics of Breast Cancer Resistance Protein
in Caco-2 Cells. Drug Metab. Dispos..

[65] Keiser M., Kaltheuner L., Wildberg C., Müller J., Grube M., Partecke L. I., Heidecke C.-D., Oswald S. (2017). The Organic
Anion-Transporting Peptide 2B1 Is Localized in the Basolateral Membrane
of the Human Jejunum and Caco-2 Monolayers. J. Pharm. Sci..

[66] Sai Y., Kaneko Y., Ito S., Mitsuoka K., Kato Y., Tamai I., Artursson P., Tsuji A. (2006). Predominant Contribution
of Organic Anion Transporting Polypeptide OATP-B (OATP2B1) to Apical
Uptake of Estrone-3-Sulfate by Human Intestinal Caco-2 Cells. Drug Metab. Dispos. Biol. Fate Chem..

[67] Kawahara I., Nishikawa S., Yamamoto A., Kono Y., Fujita T. (2020). The Impact
of Breast Cancer Resistance Protein (BCRP/ABCG2) on Drug Transport
Across Caco-2 Cell Monolayers. Drug Metab. Dispos..

[68] Yamasaki Y., Ieiri I., Kusuhara H., Sasaki T., Kimura M., Tabuchi H., Ando Y., Irie S., Ware J., Nakai Y., Higuchi S., Sugiyama Y. (2008). Pharmacogenetic Characterization
of Sulfasalazine Disposition Based on NAT2 and ABCG2 (BCRP) Gene Polymorphisms
in Humans. Clin. Pharmacol. Ther..

[69] Jacqueroux E., Hodin S., Saib S., He Z., Bin V., Delézay O., Delavenne X. (2020). Value of Quantifying
ABC Transporters
by Mass Spectrometry and Impact on *in Vitro*-to-*in Vivo* Prediction of Transporter-Mediated Drug-Drug Interactions
of Rivaroxaban. Eur. J. Pharm. Biopharm..

[70] Karlgren M., Vildhede A., Norinder U., Wisniewski J. R., Kimoto E., Lai Y., Haglund U., Artursson P. (2012). Classification
of Inhibitors of Hepatic Organic Anion Transporting Polypeptides (OATPs):
Influence of Protein Expression on Drug-Drug Interactions. J. Med. Chem..

[71] Matsson P., Pedersen J. M., Norinder U., Bergström C. A. S., Artursson P. (2009). Identification
of Novel Specific and General Inhibitors
of the Three Major Human ATP-Binding Cassette Transporters P-Gp, BCRP
and MRP2 Among Registered Drugs. Pharm. Res..

[72] EFSA European Union. EFSA J. 2010, 8 11.

[73] EFSA (2009). Scientific Opinion on the Re-Evaluation
Tartrazine (E 102). EFSA J..

[74] EFSA (2009). Scientific Opinion
on the Re-Evaluation
of Sunset Yellow FCF (E 110) as a Food Additive. EFSA J..

[75] EFSA (2009). Scientific Opinion on the Re-Evaluation
of Azorubine/Carmoisine (E 122) as a Food Additive. EFSA J..

[76] EFSA (2009). Scientific Opinion on the Re-Evaluation
of Allura Red AC (E 129) as a Food Additive. EFSA J..

[77] Kruger J., Sus N., Moser A., Scholz S., Adler G., Venturelli S., Frank J. (2024). Low β-Carotene Bioaccessibility and Bioavailability from High
Fat. Dairy-Based Meal. Eur. J. Nutr..

[78] Sy C., Gleize B., Dangles O., Landrier J.-F., Veyrat C. C., Borel P. (2012). Effects of
Physicochemical Properties of Carotenoids on Their Bioaccessibility,
Intestinal Cell Uptake, and Blood and Tissue Concentrations. Mol. Nutr. Food Res..

[79] Fang Y., Cao W., Xia M., Pan S., Xu X. (2017). Study of Structure
and Permeability Relationship of Flavonoids in Caco-2 Cells. Nutrients.

[80] Wang X., Pan Y., Jianshe M., Shi S., Zheng X., Xiang Z. (2014). Application
of a Liquid Chromatography-Tandem Mass Spectrometry Method to the
Pharmacokinetics, Bioavailability and Tissue Distribution of Neohesperidin
Dihydrochalcone in Rats. Xenobiotica.

[81] McGovern S. L., Helfand B. T., Feng B., Shoichet B. K. (2003). A Specific Mechanism
of Nonspecific Inhibition. J. Med. Chem..

[82] Nozaki Y., Izumi S. (2023). Preincubation Time-Dependent,
Long-Lasting Inhibition of Drug Transporters
and Impact on the Prediction of Drug–Drug Interactions. Drug Metab. Dispos..

[83] Sinokki A., Miinalainen A., Kiander W., Kidron H. (2024). Preincubation-Dependent
Inhibition of Organic Anion Transporting Polypeptide 2B1. Eur. J. Pharm. Sci..

[84] Izumi S., Nozaki Y., Lee W., Sugiyama Y. (2022). Experimental
and Modeling
Evidence Supporting the Trans-Inhibition Mechanism for Preincubation
Time-Dependent, Long-Lasting Inhibition of Organic Anion Transporting
Polypeptide 1B1 by Cyclosporine AS. Drug Metab.
Dispos..

[85] Tátrai P., Temesszentandrási-Ambrus C., Varga T., Gáborik Z. (2023). The Inhibitor
Preincubation Effect Is Universal to SLC Transporter Assays and Is
Only Partially Eliminated in the Presence of Extracellular Protein. Drug Metab. Dispos..

[86] Braune A., Engst W., Blaut M. (2005). Degradation
of Neohesperidin Dihydrochalcone
by Human Intestinal Bacteria. J. Agric. Food
Chem..

[87] Chung K.-T., Stevens S. E., Cerniglia C. E. (1992). The Reduction of Azo Dyes by the
Intestinal Microflora. Crit. Rev. Microbiol..

[88] Gardana C., Simonetti P., Canzi E., Zanchi R., Pietta P. (2003). Metabolism
of Stevioside and Rebaudioside A from Stevia Rebaudiana Extracts by
Human Microflora. J. Agric. Food Chem..

[89] Chan L. M.
S., Lowes S., Hirst B. H. (2004). The ABCs of Drug Transport in Intestine
and Liver: Efflux Proteins Limiting Drug Absorption and Bioavailability. Eur. J. Pharm. Sci..

[90] Takano M., Yumoto R., Murakami T. (2006). Expression
and Function of Efflux
Drug Transporters in the Intestine. Pharmacol.
Ther..

[91] Nosol K., Romane K., Irobalieva R. N., Alam A., Kowal J., Fujita N., Locher K. P. (2020). Cryo-EM
Structures Reveal Distinct
Mechanisms of Inhibition of the Human Multidrug Transporter ABCB1. Proc. Natl. Acad. Sci. U. S. A..

[92] Aller S. G., Yu J., Ward A., Weng Y., Chittaboina S., Zhuo R., Harrell P. M., Trinh Y. T., Zhang Q., Urbatsch I. L., Chang G. (2009). Structure
of P-Glycoprotein Reveals
a Molecular Basis for Poly-Specific Drug Binding. Science.

