# Supplementary material for: Food Additives Inhibit Intestinal Drug Transporters but Have Limited Effect on In Vitro Drug Permeability
Source: Mol Pharm. 2025 Aug 7;22(9):5627–37. doi: 10.1021/acs.molpharmaceut.5c00705 (PMC12406256; doi:10.1021/acs.molpharmaceut.5c00705)
Supplement: Supplementary file 1 [file mp5c00705_si_001.pdf]

SUPPORTING INFORMATION

Food additives inhibit intestinal drug transporters but have limited effect on in vitro drug permeability

Laura Suominen[a,1], Emilia Stenberg[a,1], Noora Sjöstedt[a] and Heidi Kidron[a]*

[a] Drug Research Program, Division of Pharmaceutical Biosciences, Faculty of Pharmacy, University of Helsinki, Helsinki, Finland

[1] L.S. and E.S. contributed equally to this work

* Corresponding author

Address: Heidi Kidron, P.O. Box 56 (Viikinkaari 5), FI-00014 University of Helsinki, Finland
Phone: +358 294159518
Email: heidi.kidron@helsinki.fi

Fluorescence interference studies

Fluorescence interference was studied as described in Sjöstedt et al.[1] to find out if any of the food additives cause false results by affecting the fluorescence signal (e.g., quenching fluorescence) in transporter inhibition assays. The assay was done with the fluorescent transporter substrates CDCF, LY, and 5-CF which were diluted either in 0.1 M NaOH (CDCF and 5-CF) or a 1:1 mixture of 0.1 M NaOH and 0.1 M HCl (LY samples). For each substrate, the concentration that was used was such that the baseline signal corresponded to the signal measured in the actual transport experiments. The food additives were added to the substrate solutions at the maximal achievable concentration reached in the assay assuming that 100% of the test compound is present in the final eluate in the experiment. The fluorescence was measured with the same parameters as in the vesicle and OATP2B1 assays. Potential inhibiting test compounds that caused interference to the fluorescence were also studied with lower concentrations, 50%, 20%, and 10% of the maximal achievable concentration.

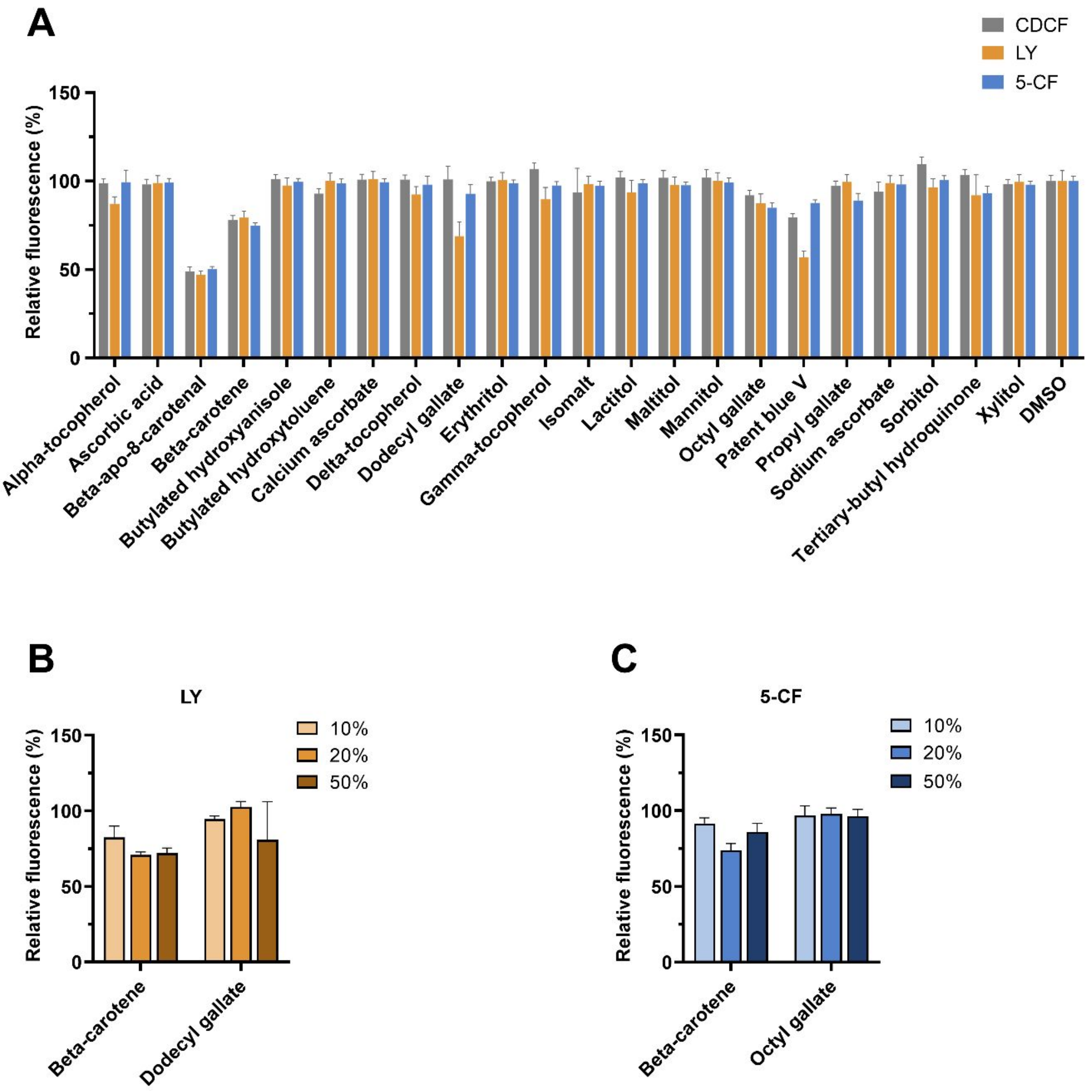

Figure S1. *Fluorescence interference of the food additives with 5(6)-carboxy-2,7-dichlorofluorescein (CDCF), Lucifer yellow (LY), and 5-carboxyfluorescein (5-CF). Fluorescent measurement was done in conditions mimicking the final solution in transport assays and assuming that 100% (A) or 10 – 50% (B and C) of the test compound is present. Data (mean ± SD) is presented as relative fluorescence normalized to the control (DMSO, 100%).*

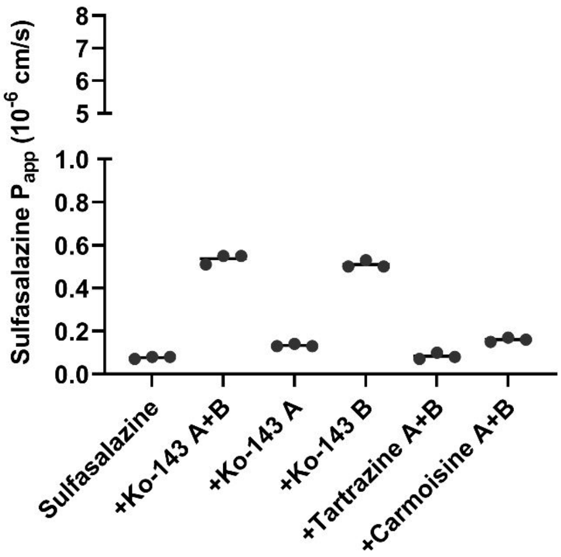

Figure S2. *Sulfasalazine permeability in Caco-2 cells in the presence of Ko-143, tartrazine, or carmoisine. Inhibition potential of Ko-143 on different sides of the Caco-2 monolayer was studied by measuring the apparent permeability (P_{app}) of sulfasalazine. Ko-143 (10 µM) was added on the apical (A), basolateral (B), or on both (A+B) sides of Caco-2 cells. Tartrazine and carmoisine (200 µM) were added on both sides, and sulfasalazine (500 µM) permeability was measured in apical to basolateral direction. DMSO concentration was 0.45%. Circles present P_{app} from three replicate wells and lines show the mean.*

REFERENCES

(1) Sjöstedt, N.; Deng, F.; Rauvala, O.; Tepponen, T.; Kidron, H. Interaction of Food Additives with Intestinal Efflux Transporters. *Molecular Pharmaceutics* **2017**, *14* (11), 3824–3833.
